# Miscellaneous

**Published:** 1889-09

**Authors:** 


### MISCELLANEOUS.

Milk and Powdered Elm Bark.—The fact should be known that finely powdered elm bark, say two teaspoonfuls to a pint of new milk, will prevent coagulation in the itomach of children of milk into hard, tough lumps, so that the milk will be more readily digested.

To Stop Coughing.—Coughing and sneezing can be stopped by pressing on the nerves of the lips in the neighborhood of the nose.

Sugar in Hiccough in Children.—Dr. H. Tucker (*Southern Medical Journal*) says he has never known the following treatment to fail in hiccough: moisten granulated sugar with good cider vinegar; give to an infant from a few grains to a teaspoonful. The effect is almost instantaneous, and the dose seldom needs to be repeated.

Erysipelas.—The *Medical Record* states that the sticking Plaster Treatment of Erysipelas is highly recommended by Professor Wolfler of Gratz. Strips of isinglass plaster about the breadth of the thumb, are applied over the affected surface.

A General Antidote for Poisons.—According to the *American Journal of Pharmacy*, a general antidote for poisons may be made by mixing equal parts of calcined magnesia, wood charcoal' and hydrated oxide of iron, and is applicable in cases in which the poison is unknown. It should not, of course, supersede the stomach pump or other forms of emesis.

Mr. Ruskin speaking of a certain tribe of bees, says, "they will steal each other's nests like human beings and fight like Christians."

We all Know—hearing so many fulsome reports about the matter—how much money is usually raised in this country by Protestant Missionary Societies, for the conversion of the heathen. Statistics on the subject show that it costs enormously, in proportion to the questionable good effected. We quote from a late report, as follows:

Last year, in Ceylon, 424 agents of the Church Missionary Society spent over $55,000 in making 190 adult converts out of a population of nearly three millions; but the relapses were more numerous than the converts, as there was a decrease of 143 native Christian adherents. In China 247 agents of the same society spent $74,375 in making 116 converts out of a population of 382,000,000. In Northern India (Bengal, Bombay and the Northwest provinces) 715 agents made 173 convert at a cost of $170,930.

Grave-Yard Pestilence.—Sir Spencer Wells, the eminent English surgeon, at a recent meeting of the Scottish Burial Reform and Cremation Society, called attention to the danger of the extension of disease through grave-yards. He mentioned one cemetery in this vicinity of London in which nine thousand persons are buried yearly, and cited a remarkable case which occurred in Yorkshire as an illustration of the propagation of specific disease through grave-yard infection. Several scarlet-fever patients were buried in a church-yard. A portion of the church-yard was afterward included in the garden of the rector, who had it dug up.

Scarlet fever broke out in the household of the rector, and in a number of families in the neighborhood. It seems incredible that the germs of this disease should survive so long an exposure to the disintegrating elements, but the story is vouched for by a man whose integrity is not to be impeached.

VERSE-WRITING.—"In our own immediate times verse-writing has become something more of the nature of a disease than of an honor. A species of rhymophobia pervades the cultivated world. Like the bite of the bitten victim, fashionable forms of construction extend. There is contagion in them. The strain for effect has become virulent. We feel, perforce, a sympathy with the half-playful but wholly earnest revolt of Dr. Holmes against the epidemic character of our debilitated verse.

"That overbalanced struggle for perfection of manner which stifles the spirit; the renaissance of obsolete forms which vitiates the modernness of sympathy so necessary to healthful work; the endless tricking and decking of little thoughts; the apparant unconsciousness of whether one's thought be large or little, or whether it be worth thinking of at all, or if worth thinking, whether worth thinking in poetry—these qualities characterize so much of the verse of our day that one may be pardoned for becoming more aware of them than of some other and better traits which undoubtedly accompany them. It may be said that there is a certain loss of the sense of proportion in our poetic power. By this I mean that higher proportion which is to proportion of form as the soul is to the human body. We do not build loftily. We do not live to last. We do not always know why we build at all. The result is a lack of architecture. But we have plenty of verse-carpentering; done as neatly as the service of Adam Bede, who thought the world was to be saved by conscientious day's labor. But the paper cap of the workman looks over the whole job."—*Miss Phelps in* THE CENTURY.

ON A DROOPING BOUGH of a large elm, close by a hotel in Sunderland, Mass., two English robins have made a nest. Strong winds caused so much swaying as to endanger the eggs in the nest. The birds have been equal to the emergency. They have secured some twine and fastened one end under the nest and the other end to the larger branch below, thus avoiding the danger of too much oscillation. The instinct exhibited by these birds has attracted considerable attention.

---

THE death rate on the earth is calculated to be sixty-seven in a minute, or 4,020 an hour, 96,480 a day, 35,215,200 a year. The birth rate slightly exceeds this. It is calculated to be seventy per minute, 4,200 in an hour, 100,800 a day, or 36,742,000 in a year. The estimated increase per annum, according to this is, therefore, only a little more than 1,500,000, and it will be many centuries before our earth gets to be so crowded that the inhabitants will jostle each other over the edge into space. The Malthusian's ideas regarding over-population need not, therefore, detain any one from marriage.

